# Refractory Diabetic Ketoacidosis Associated with Enfortumab Vedotin: A Pharmacokinetic-Based Management Approach

**DOI:** 10.34067/KID.0000001262

**Published:** 2026-06-15

**Authors:** Shivangi K. Patel, Taylor Purzycki, Alaa Mahmoud, John Saldarini

**Affiliations:** 1 Department of Nephrology, Morristown Medical Center, Morristown, New Jersey; 2 Department of Internal Medicine, Morristown Medical Center, Morristown, New Jersey

**Keywords:** acidosis, cancer

## Abstract

Enfortumab vedotin (EV), an antibody-drug conjugate approved for metastatic urothelial carcinoma, is increasingly recognized to cause severe hyperglycemia and diabetic ketoacidosis (DKA). However, optimal management of EV-associated refractory DKA remains undefined. We describe a 57-year-old man receiving EV and pembrolizumab who developed profound insulin-resistant DKA that persisted despite standard therapy. EV composed of a nectin-4–targeted monoclonal antibody linked to the cytotoxic agent monomethyl auristatin E (MMAE), which is the primary cause of systemic toxicity. Systemic elimination of MMAE is *via* metabolism by cytochrome P450 3A4 and biliary/fecal excretion. Tissue exposure is limited *via* P-glycoprotein efflux pump. Therefore, in the setting of refractory DKA, a pharmacologic enzyme induction was initiated to mitigate further MMAE-induced injury. After initiation of induction alongside continued insulin therapy, resolution of ketoacidosis occurred within 46 hours. This case illustrates a pharmacokinetic-informed approach to EV-associated refractory DKA and highlights how understanding drug metabolism may improve therapeutic outcomes when conventional therapy is insufficient.

## Introduction

Enfortumab vedotin (EV) is an antibody-drug conjugate targeting nectin-4 that is conjugated to the microtubule disrupting agent, monomethyl auristatin E (MMAE).^1^ In combination with pembrolizumab, EV is approved as first-line therapy for locally advanced or metastatic urothelial carcinoma.^2^ Although EV improves survival outcomes, it has been increasingly associated with severe hyperglycemia and diabetic ketoacidosis (DKA), which may be refractory to standard treatment.^1,3,4^ Both EV and pembrolizumab (immune checkpoint inhibitor [ICI]) have been implicated in DKA.^1,3–5^ However, ICI-associated diabetes is typically characterized by autoimmune *β*-cell destruction developing over weeks to months, resulting in permanent insulin dependence.^5^ By contrast, EV-associated DKA appears to reflect profound insulin resistance with preserved endogenous insulin secretion. Optimal management strategies for severe or persistent cases remain undefined. We describe a case of refractory EV-associated DKA managed using a pharmacokinetic-informed strategy aimed at mitigating ongoing drug-related ketoacidosis.

## Case Description

A 57-year-old man with metastatic urothelial carcinoma presented 1 week after cycle 1, day 8 of EV (120 mg; maximum 125 mg), and pembrolizumab with nausea, vomiting, and poor oral intake. He had no history of diabetes and no exposure to CYP3A4-inhibiting drugs or foods. His body mass index (BMI) was 30.5 kg/m^2^.

Initial laboratory results: creatinine 0.75 mg/dl, glucose >300 mg/dl range, bicarbonate 8 mmol/L, albumin 2.1 g/dl with corrected anion gap 22.8 mEq/L (calculated anion gap+ 2.5×[4.0−albumin]), arterial pH 7.30, and *β*-hydroxybutyrate (BHB) >46 mg/dl, lactate approximately 2–3 mmol/L, glucosuria and ketonuria. Evaluation of hyperglycemia revealed hemoglobin A1c 5.9%, negative testing for glutamic acid decarboxylase-65 and zinc transporter-8 antibodies, and elevated C-peptide of 8.9 ng/ml, consistent with preserved endogenous insulin secretion. Blood and urine cultures were negative. Imaging demonstrated no new findings. Hemodynamics remained stable. The clinical picture was consistent with drug-induced DKA.

Standard DKA therapy consisting of 5% dextrose in lactated Ringer solution with potassium chloride, aggressive intravenous fluids (up to approximately 6 L/d), and continuous insulin was initiated. Despite insulin infusions >0.55 U/kg per hour, BHB remained >46 mg/dl. Daily insulin requirements peaked at 1421 units (Figure 1). Insulin-induced electrolyte abnormalities developed, including hypophosphatemia (0.6 mg/dl), hypomagnesemia (1.4 mg/dl), and hypokalemia (nadir 3.0 mmol/L). Aggressive potassium repletion with intravenous potassium chloride administered *via* maintenance fluids and intermittent boluses was required to maintain serum potassium above 3.5 mmol/L. Despite intervention, the patient remained unstable and developed worsening confusion resulting in transfer to the intensive care unit. His vital signs were temperature 36.7**°C**, BP 115/71 mm Hg, heart rate 99 beats/min, respiratory rate 30 breaths/min, and oxygen saturation 98% on room air. Venous blood gas showed pH 7.32 with Pco_2_ 16 mm Hg. Noncontrast computed tomography of the brain demonstrated no acute intracranial pathology. Electroencephalography was not performed, as there were no focal neurologic deficits or seizure activity. Although acidemia was modest, the severity and persistence of ketosis with extreme insulin resistance was felt to be the primary driver of encephalopathy.

**Figure 1:**
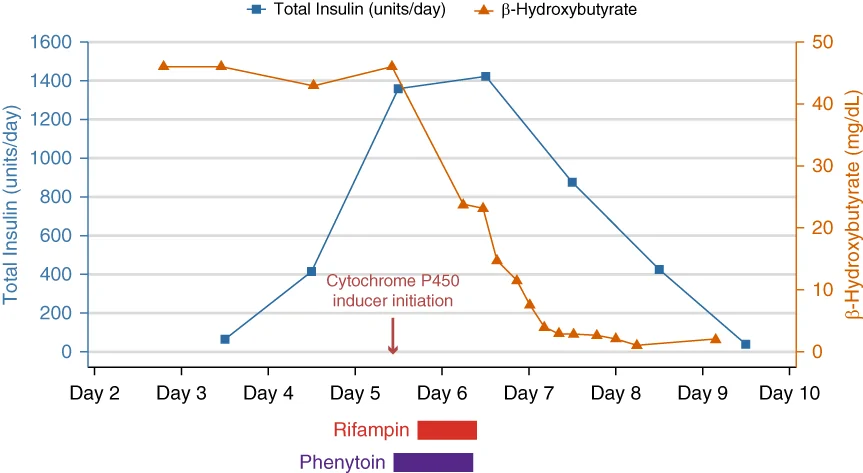
**Trend in serum BHB levels and daily insulin infusion requirements after initiation of CYP3A4/P-gp induction.** BHB, *β*-hydroxybutyrate; CYP3A4, P-gp, P-glycoprotein.

Given the temporal relationship of DKA to the EV and preserved endogenous insulin secretion, EV-associated metabolic toxicity was suspected. Because MMAE is predominantly metabolized by CYP3A4 and excreted *via* P-glycoprotein (P-gp), pharmacologic induction of these proteins was pursued. Phenytoin (200 mg oral three times daily) was started, followed 6 hours later by rifampin (600 mg oral daily).

BHB had shown minimal improvement with >72 hours standard therapy. Within 24 hours of starting phenytoin and rifampin, BHB fell from >20 to <5 mg/dl, insulin requirements declined (Figure 1), and lactate and encephalopathy resolved. Phenytoin level was 3.6 ug/ml 24 hours later after a total of 1 g (five doses); a therapeutic anticonvulsant level was not targeted, as phenytoin was used solely for enzyme induction. He transitioned to subcutaneous insulin and ultimately weaned off insulin entirely without DKA recurrence.

## Discussion

This patient presented with DKA while receiving treatment with pembrolizumab and EV. Pembrolizumab-induced autoimmune diabetes was initially considered given its well-known and better characterized association with DKA. However, the normal hemoglobin A1c, negative pancreatic autoantibodies, and elevated C-peptide levels indicating preserved endogenous insulin secretion, argued against this diagnosis.^5^ EV-associated DKA was then considered.^1,3,4^

Hyperglycemia occurs in approximately 17% of EV-treated patients, with approximately 7% requiring insulin therapy and a subset developing life-threating DKA.^1^ In contrast to ICI-associated diabetes, EV-associated hyperglycemia is reversible, suggesting a functional rather than destructive mechanism.^3–5^ While the mechanism is unknown, similar severe but reversible episodes of insulin resistance with preserved *β*-cell function have been reported with the PI3K-*α* inhibitor alpelisib.^6^ Disruption of PI3K/AKT (also known as protein kinase B) signaling may therefore provide a plausible mechanism for EV-associated insulin resistance.

EV is a highly efficient delivery vehicle for its cytotoxic compound, MMAE.^1,2^ Once released, free MMAE is highly protein bound (68% to 82%) in plasma and lipophilic, easily permeating cell membranes resulting in large volume of distribution.^1^ MMAE can accumulate in insulin-sensitive tissues such as liver, muscle, and adipose tissue.^7^ In the liver, reduction of insulin-induced AKT activation results in continued gluconeogenesis and reduced glycogen synthesis. In adipose tissue, AKT inhibition results in continued lipolysis and free fatty acid delivery to the liver, enhancing ketone production. In addition, in peripheral tissues including adipose and muscle, MMAE disrupts the microtubular scaffolding necessary for glucose transporter type 4 vesicular transport to cell surfaces, limiting glucose transport from blood to cells. Collectively, these changes result in progressive hyperglycemia and DKA.^6–8^

The lipophilic nature of MMAE may explain why the duration of effect appears longer than the plasma t_1/2_ (approximately 2.6 days). It may also explain the variability observed in EV-associated DKA severity and response to treatment.^1,4,5^ Prior case reports documented treatment with dialysis or therapeutic plasma exchange with limited effect.^4,9^ The highly protein-bound nature of MMAE makes dialysis an inefficient mechanism of drug removal. While therapeutic plasma exchange is expected to remove protein-bound drug in the plasma, free MMAE’s large volume of distribution would lead to rapid rebound and limited net effect.^4^ Effectiveness would be further reduced in patients with larger BMI, as in this case, due to higher EV dosing and larger volume of distribution. Both a BMI >30 kg/m^2^ and increased systemic drug exposure are risk factors for EV-associated DKA.^1^ Given these considerations, and assuming intracellular persistence of MMAE was the primary cause of DKA in this patient, targeting mechanisms to enhance MMAE elimination were considered a plausible therapeutic intervention.

Systemic elimination of free MMAE occurs *via* hepatic CYP3A4-mediated metabolism and biliary/fecal excretion (approximately 72% recovered in stool), with minimal renal clearance. Removal from peripheral tissue is dependent on P-gp, an efflux transporter, which serves to limit intracellular exposure to the compound.^1,10^ Consistent with this understanding, rifampin, a strong inducer of the CYP3A4 and P-gp efflux transporter, has been shown to rapidly reduce levels of free MMAE; coadministration with brentuximab vedotin (which shares the same MMAE payload) reduced MMAE area under the curve by approximately 46%, and a physiologically based pharmacokinetic model for EV predicted a similar approximately 53% reduction in MMAE exposure.^1,10–12^

Pharmacologic induction of CYP3A4 and P-gp for treatment of tacrolimus toxicity has been well documented, with both rifampin and phenytoin used.^13,14^ Phenytoin was initiated for worsening encephalopathy for its central nervous system benefits, with the additional advantage of CYP3A4 induction. Rifampin was added to augment induction based on existing pharmacologic data.^1,10–12,14^ Serial BHB and insulin requirements served as surrogate markers of ongoing MMAE-related DKA. After drug initiation, the patient showed a clear shift in metabolic trajectory, with decreasing ketonemia and rapidly falling insulin requirements. Although spontaneous recovery cannot be excluded, the temporal pattern supports attenuation of MMAE-related effects after enzyme induction.

Most published cases of EV-associated DKA involve prolonged critical illness, multiorgan failure, or death. Handful of surviving cases, including this one, demonstrate preserved C-peptide levels and ultimately reversible insulin resistance, distinguishing them from ICI-associated diabetes.^3–5^ We believe the successful outcome in this case provides evidence for targeted use of CYP3A4 and P-gp inducers to limit duration of MMAE toxicity when treatment appears refractory to standard therapies in the setting of life-threatening DKA.

## Supplementary Material

## Data Availability

All original data, including deidentified patient-level data or individual laboratory data measurements, are included in the manuscript and/or supplemental material.

## Declarative Statements

This study includes clinical experimentation and received Institutional Review Board or Ethics Committee approval. All patients provided written informed consent.
