# Supplementary material for: Refractory Diabetic Ketoacidosis Associated with Enfortumab Vedotin: A Pharmacokinetic-Based Management Approach
Source: Kidney360. 2026 Jun 15;7(9):2131–3. doi: 10.34067/KID.0000001262 (PMC13641496; doi:10.34067/KID.0000001262)
Supplement: Supplementary file 1 [file kidney360-7-2131-s001.pdf]

ASN American Society of Nephrology
JASN Journal of the American Society of Nephrology
KIDNEY360 Accessing Our World From Every Angle
CJASN Clinical Journal of the American Society of Nephrology

# ASN Journal Disclosure Form

As per ASN journal policy, I have disclosed any financial relationships or commitments I have held in the past 36 months as included below. I have listed my Current Employer below to indicate there is a relationship requiring disclosure. If no relationship exists, my Current Employer is not listed.

A. Mahmoud reports the following:
Employer: Morristown Medical Center

I understand that the information above will be published within the journal article, if accepted, and that failure to comply and/or to accurately and completely report the potential financial conflicts of interest could lead to the following: 1) Prior to publication, article rejection, or 2) Post-publication, sanctions ranging from, but not limited to, issuing a correction, reporting the inaccurate information to the authors' institution, banning authors from submitting work to ASN journals for varying lengths of time, and/or retraction of the published work.

Name: Alaa Mahmoud
Manuscript ID: K360-2026-000286
Manuscript Title: Refractory Diabetic Ketoacidosis Associated with Enfortumab Vedotin: A Pharmacokinetic-Based Management Approach
Date of Completion: April 12, 2026
Disclosure Updated Date: April 12, 2026

ASN
American Society of Nephrology
JASN
Journal of the American Society of Nephrology
KIDNEY360
Accessing Our World From Every Angle
CJASN
Clinical Journal of the American Society of Nephrology

# ASN Journal Disclosure Form

As per ASN journal policy, I have disclosed any financial relationships or commitments I have held in the past 36 months as included below. I have listed my Current Employer below to indicate there is a relationship requiring disclosure. If no relationship exists, my Current Employer is not listed.

S. Patel reports the following:
Employer: Atlantic Health System

I understand that the information above will be published within the journal article, if accepted, and that failure to comply and/or to accurately and completely report the potential financial conflicts of interest could lead to the following: 1) Prior to publication, article rejection, or 2) Post-publication, sanctions ranging from, but not limited to, issuing a correction, reporting the inaccurate information to the authors' institution, banning authors from submitting work to ASN journals for varying lengths of time, and/or retraction of the published work.

Name: Shivangi Patel
Manuscript ID: K360-2026-000286R1
Manuscript Title: Refractory Diabetic Ketoacidosis Associated with Enfortumab Vedotin: A Pharmacokinetic-Based Management Approach
Date of Completion: April 25, 2026
Disclosure Updated Date: April 25, 2026

ASN
American Society of Nephrology
JASN
Journal of the American Society of Nephrology
KIDNEY360
Accessing Our World From Every Angle
CJASN
Clinical Journal of the American Society of Nephrology

# ASN Journal Disclosure Form

As per ASN journal policy, I have disclosed any financial relationships or commitments I have held in the past 36 months as included below. I have listed my Current Employer below to indicate there is a relationship requiring disclosure. If no relationship exists, my Current Employer is not listed.

T. Purzycki has nothing to disclose.

I understand that the information above will be published within the journal article, if accepted, and that failure to comply and/or to accurately and completely report the potential financial conflicts of interest could lead to the following: 1) Prior to publication, article rejection, or 2) Post-publication, sanctions ranging from, but not limited to, issuing a correction, reporting the inaccurate information to the authors' institution, banning authors from submitting work to ASN journals for varying lengths of time, and/or retraction of the published work.

Name: Taylor Purzycki
Manuscript ID: K360-2026-000286R1
Manuscript Title: Refractory Diabetic Ketoacidosis Associated with Enfortumab Vedotin: A Pharmacokinetic-Based Management Approach
Date of Completion: May 13, 2026
Disclosure Updated Date: April 13, 2026

ASN
American Society of Nephrology
JASN
Journal of the American Society of Nephrology
KIDNEY360
Accessing Our World From Every Angle
CJASN
Clinical Journal of the American Society of Nephrology

# ASN Journal Disclosure Form

As per ASN journal policy, I have disclosed any financial relationships or commitments I have held in the past 36 months as included below. I have listed my Current Employer below to indicate there is a relationship requiring disclosure. If no relationship exists, my Current Employer is not listed.

J. Saldarini has nothing to disclose.

I understand that the information above will be published within the journal article, if accepted, and that failure to comply and/or to accurately and completely report the potential financial conflicts of interest could lead to the following: 1) Prior to publication, article rejection, or 2) Post-publication, sanctions ranging from, but not limited to, issuing a correction, reporting the inaccurate information to the authors' institution, banning authors from submitting work to ASN journals for varying lengths of time, and/or retraction of the published work.

Name: John C. Saldarini
Manuscript ID: K360-2026-000286R1
Manuscript Title: Refractory Diabetic Ketoacidosis Associated with Enfortumab Vedotin: A Pharmacokinetic-Based Management Approach
Date of Completion: April 26, 2026
Disclosure Updated Date: April 26, 2026
